# Supplementary material for: Single Cell Transcriptomic and Chromatin Profiles Suggest Layer Vb Is the Only Layer With Shared Excitatory Cell Types in the Medial and Lateral Entorhinal Cortex
Source: Front Neural Circuits. 2022 Jan 26;15:806154. doi: 10.3389/fncir.2021.806154 (PMC8826650; doi:10.3389/fncir.2021.806154)
Supplement: Supplementary file 14 [file Data_Sheet_1.ZIP › Sup_vs_Deep_Motifs/homerResults/motif26.similar.html]

motif26

## Information for motif26

A
C
G
T
A
G
T
C
A
C
G
T
A
C
T
G
A
C
T
G
A
C
T
G
A
C
T
G
A
G
T
C
A
G
T
C
A
C
G
T
  
Reverse Opposite:  

C
G
T
A
A
C
T
G
A
C
T
G
A
G
T
C
A
G
T
C
A
G
T
C
A
G
T
C
C
G
T
A
A
C
T
G
C
G
T
A
  

|  |  |
| --- | --- |
| p-value: | 1e-10 |
| log p-value: | -2.364e+01 |
| Information Content per bp: | 1.530 |
| Number of Target Sequences with motif | 68.0 |
| Percentage of Target Sequences with motif | 0.20% |
| Number of Background Sequences with motif | 7.4 |
| Percentage of Background Sequences with motif | 0.09% |
| Average Position of motif in Targets | 318.0 +/- 254.7bp |
| Average Position of motif in Background | 476.0 +/- 393.0bp |
| Strand Bias (log2 ratio + to - strand density) | -0.0 |
| Multiplicity (# of sites on avg that occur together) | 1.00 |
| Motif File: | file (matrix) reverse opposite |

### Similar de novo motifs found

|  |  |  |  |  |  |  |  |
| --- | --- | --- | --- | --- | --- | --- | --- |
| Rank | Match Score | Redundant Motif | P-value | log P-value | % of Targets | % of Background | Motif file |
